# Exploration of Natural Protein–Polysaccharide–Polyphenol Ternary Complexes from Grape Pomace for Clean-Label Pickering Emulsions Through pH Adjustment

**DOI:** 10.3390/foods15030564

**Published:** 2026-02-05

**Authors:** Zixuan Du, Zhengyang Jia, Jianyu Yang, Yue Zhao, Jiachen Zang, Guanghua Zhao

**Affiliations:** 1 College of Food Science and Nutritional Engineering, China Agricultural University, Beijing 100083, China; tshuadzx@163.com; 2 China Agricultural University-Sichuan Advanced Agricultural & Industrial Institute, Chengdu 611430, China; 3 College of Food Science, Northeast Agricultural University, Harbin 150030, China; 4 Yangtze River Delta Smart Oasis Innovation Center, Zhejiang University, Jiaxing 314102, China

**Keywords:** grape pomace, ternary complex, Pickering emulsion, pH

## Abstract

Grape pomace represents a major organic solid waste generated by the wine industry, but its application has been largely unexplored. On the other hand, so far, stable and sustainable raw materials for producing stable, edible Pickering emulsifiers suitable for the food industry have been lacking. To solve these problems, this study established a mild but effective co-extraction method to obtain protein–polysaccharide–polyphenol ternary complexes (GPTCs) from grape pomace. Subsequently, these complexes were directly developed into an edible Pickering emulsifier by a pH-controlled method. Results showed that a series of properties related to the Pickering emulsifier, such as particle size, surface charge, wettability, and interfacial adsorption behavior, could be easily controlled by adjusting the solution’s pH. Consequently, the GPTC prepared at pH 7.0 exhibited optimal emulsifying performance. The resulting particles had an average particle size of approximately 111 nm, and stabilized Pickering emulsions with a volume-weighted mean oil droplet diameter (D [4,3]) of 9.49 μm, indicating high emulsion stability. Collectively, this study provided an actionable approach for the green, high-value utilization of wine byproducts by establishing a pH-responsive design framework for edible Pickering emulsifiers.

## 1. Introduction

Driven by long-term population growth and sustained global demand, the wine industry has maintained a large-scale production, resulting in the substantial and persistent generation of organic solid waste. It has been reported that the wine sector represents the largest contributor to organic solid waste [1]. Statistics indicate that every 750 L of wine produced generates up to 200 kg of organic solid waste [2], approximately 60% of which consists of a mixture of grape skins and seeds. This solid waste is known as grape pomace. Current techno-economic studies propose multiple value-added solutions for grape pomace. The polyphenols and other organic compounds within grape pomace can be utilized to produce high-value products such as food additives or pharmaceuticals [3], or to manufacture added-value products like fertilizers [4], biofuels [5], and animal feed.

So far, studies related to high-value utilization of grape pomace in the wine industry have been mainly focused on grape seed protein. In the context of wine processing, studies have proposed using grape seed protein and its hydrolysates as natural color stabilizers or clarification agents in post-fermentation wine liquors, partially replacing traditional animal-derived or exogenous protein clarifiers and thereby improving wine stability and enhancing sustainability [6]. In addition, within the feed industry, grape seed protein and related extracts are being explored as functional protein ingredients or additives for ruminant feed systems [7]. Recently, it has been reported that the protein purity and functional properties have been enhanced by controlling the extraction pH, temperature, and solvent conditions [8].

On the other hand, polyphenols can also be efficiently extracted from grape pomace using aqueous or ethanol-based solvents [9]. These grape pomace-derived polyphenols exhibit strong antioxidant activity and have been widely proposed as synthetic antioxidant replacers. Owing to their bioactivity, they can be applied in various food systems to inhibit microbial spoilage, reduce lipid oxidation, and ultimately prolong the shelf life of food products. Regarding the carbohydrate fraction in grape pomace, as reported by Jin and colleagues, reported a comprehensive compositional characterization of industrial white and red grape pomaces and proposed that the carbohydrate-rich components could be purified and further converted into uronic acids. These uronic acid-based derivatives were suggested to have potential applications in the cosmetic and plastic industries [10].

Meanwhile, research on Pickering emulsions has increasingly been focused on edibility and renewability. From a food science perspective, such biomass-derived particles not only require sufficient interfacial activity but must also meet the edibility and cost-effectiveness requirements demanded by the food industry. To date, biomass-based natural pellets have not yet been widely available on the market, and their scale and price are not suitable for general industrial applications. Therefore, the separation, generation, and production of suitable pellets using natural biopolymer materials is of great importance, but has been largely unexplored [11].

In recent years, pH has frequently been employed as a key variable for tuning Pickering emulsion stabilizers, because variations in pH can induce changes in protein higher-order structures and alter the charge distribution of individual components within the complexes [12,13]. Previous studies have often relied on electrostatic interactions among different constituents to drive the assembly or complexation of stabilizing particles [11,14]. However, existing pH-related studies predominantly focus on purified or reconstituted biopolymers, with limited attention paid to the use of pH to regulate naturally sourced multicomponent systems. In such multicomponent systems, the role of pH in governing the formation and interfacial properties of particles remains unexplored.

Against this backdrop, this study aims to establish a scalable, reproducible mild co-extraction strategy to develop a method for large-scale extraction of a ternary complex comprising proteins, polysaccharides, and polyphenols from grape pomace. The research focuses on determining how the primary variable, pH, influences the particle size of the complex within a specific formulation space, thereby affecting its interfacial properties. This work lays the foundation for transforming grape pomace into a functional clean-label food ingredient.

## 2. Materials and Methods

### 2.1. Materials

For the preparation of grape seed ternary complexes, red grape pomace derived from *Vitis vinifera* cv. Cabernet Sauvignon, harvested in Korla, Xinjiang, was obtained from Guiji Winery Co., Ltd. (Heshuo County, Bayingolin Mongol Autonomous Prefecture, Xinjiang, China), with a high medium-chain triglyceride content (MCT ≥ 99%, Aladdin Biochemical Technology Co., Ltd., Shanghai, China). All chemicals were of analytical grade.

### 2.2. Characterization of Different pH Levels of Grape Pomace Ternary Complex (GPTC)

#### 2.2.1. Extraction of Grape Seed Ternary Complex and Preparation of GPTC at Different pH Levels

Dried grape pomace was milled with a ball mill (MM 400, Retsch GmbH, Haan, Germany) at 30 Hz for 2 min. Lipids were removed by hexane extraction (1:15 *w*/*v*) under stirring at 25 °C for 3 h, repeated three times. The defatted grape seed particles were then passed through an 80-mesh stainless-steel sieve. The sieved material was soaked in deionized water (1:10, *w*/*v*) and adjusted to pH 10.0 with 1 M NaOH. The suspension was stirred at 25 °C for 1 h, followed by ultrasonication at 100 W for 1 h, and centrifuged at 5393× *g* for 30 min; the supernatant was retained. The supernatant was adjusted to pH 3.8 with 1 M HCl to induce precipitation. The suspension was centrifuged at 5393× *g* for 30 min. The precipitate was then collected, freeze-dried, and stored at −20 °C.

The prepared grape seed ternary complex was dissolved in deionized water at 2% (*w*/*v*). Under continuous stirring, the pH was adjusted with 1 M NaOH to pH 7.0, 8.0, 9.0, and 10.0, yielding samples named GPTC-pH 7, GPTC-pH 8, GPTC-pH 9, and GPTC-pH 10. The GPTC-pH 7 sample was stirred at 25 °C for 1 h and then adjusted to pH 10.0 with 1 M NaOH, this sample was named GPTC-pH 10-c.

#### 2.2.2. Chemical Composition Analysis of GPTC

The ternary protein–polysaccharide–phenolic complexes (GPTCs) were analyzed for proximate composition. Protein and ash contents were determined following AOAC standard methods [15]: The micro-Kjeldahl method for nitrogen determination (960.52) and ash content by ignition in a furnace at 525 °C for 5 h (942.05), respectively. The percentage of crude protein was calculated by multiplying the nitrogen content by a factor of 6.25. Total carbohydrate content was measured by the phenol–sulphuric acid method [16], using glucose as standard. Total phenolic content was determined using the Folin–Ciocalteu method, with gallic acid as standard, and the results were expressed as mg gallic acid equivalents (GAEs) per gram of sample [17].

#### 2.2.3. Fourier Transform Infrared Spectroscopy (FT-IR)

Samples were analyzed using an FT-IR spectrometer (Nicolet 6700, Nicolet Corp., Madison, WI, USA). Spectra were collected over 400–4000 cm^−1^ with 32 scans. Data processing was performed in Omnic software (v9.2, Thermo Nicolet, Madison, WI, USA).

#### 2.2.4. Thermal Analysis

Thermogravimetric analysis (TGA) was conducted using an STA200 simultaneous thermal analyzer (Hitachi High-Tech Science Corp., Tokyo, Japan). Approximately 10 mg of sample was heated from 30 °C to 500 °C at 10 °C/min under nitrogen (50 mL/min).

#### 2.2.5. Particle Size and Zeta Potential

The average particle size and zeta potential of all samples were determined at 25 °C using a Malvern Zetasizer Nano ZS90 (Malvern Instruments Ltd., Malvern, Worcestershire, UK). Measurements were performed in triplicate with freshly prepared samples.

#### 2.2.6. Emulsifying Activity Index (EAI) and Emulsion Stability Index (ESI) of Emulsifiers

The emulsifier solutions (GPTC-pH 7, GPTC-pH 8, GPTC-pH 9, GPTC-pH 10, GPTC-pH 10-c) were prepared at 2% (*w*/*v*). To form the emulsions, 6.0 mL of emulsifier solution was mixed with 4 mL of MCT in a 50 mL centrifuge tube and homogenized (T18 basic, IKA, Staufen, Germany) at 10,000 rpm for 2 min. Aliquots (50 μL) were withdrawn at 0 min and 30 min and immediately diluted into 5.0 mL of 0.1% (*w*/*v*) sodium dodecyl sulfate. Absorbance at 500 nm was measured in a 1 cm cuvette. The emulsifying activity index (EAI) and emulsion stability index (ESI) were calculated as follows:

$$\mathrm{EAI}(m^{2}/g)\;=\;\frac{2\;\times \;2.303\;\times \;A_{0\;}\times \;DF}{C\;\times \;\varphi \;\times \;10^{4}}$$

$$\mathrm{ESI}(\min)=\frac{{Absorbance}_{0}}{{Absorbance}_{0\;}-\;{Absorbance}_{30}}$$

With the variables defined below:

*Absorbance*_0_: Absorbance at 500 nm immediately after dilution (0 min).

*Absorbance*_30_*:* Absorbance at 500 nm after 30 min (A at time Δt = 30 min).

*DF*: Dilution factor (5.0 mL/0.05 mL = 100).

*C*: Emulsifier concentration in the aqueous phase, g/mL (2% *w/v* = 0.02 g/mL).

*φ*: Oil volume fraction during emulsification (4/(6 + 4) = 0.4).

10^4^: Unit conversion from cm^2^ to m^2^ (1 m^2^ = 10^4^ cm^2^).

### 2.3. Emulsifying Performance of GPTC at Different pH Levels

#### 2.3.1. Emulsion Preparation

The aqueous phase was prepared using 2% (*w*/*v*) solutions of GPTC-pH 7, GPTC-pH 8, GPTC-pH 9, GPTC-pH 10, GPTC-pH 10-c, while MCT served as the oil phase at 40% (*v*/*v*). The two phases were homogenized using a high-speed disperser (T18 basic, IKA) at 10,000 rpm for 2 min to form emulsions. Fresh emulsions were immediately transferred into airtight glass vials for characterization.

#### 2.3.2. Emulsion Droplet Size

Emulsion droplet size distributions were analyzed by laser diffraction (Mastersizer 3000, Malvern Instruments Ltd., Malvern, Worcestershire, UK). Triplicate measurements were conducted for each sample.

#### 2.3.3. Confocal Laser Scanning Microscopy (CLSM)

Emulsions prepared with a ternary complex concentration of 2.0 wt% relative to the aqueous phase (φ = 0.4) were dual-stained with 0.1% Fluorescein Isothiocyanate (excitation 495 nm) to visualize polysaccharide/protein structure and 0.1% Nile Red (excitation 555 nm) to label MCT. After thorough mixing for 60 s, microstructures were examined by confocal laser scanning microscopy (A1R HD25, Nikon Corporation, Tokyo, Japan).

#### 2.3.4. Optical Microscopy

A 20 μL aliquot of each emulsion was placed on a microscope slide, covered with a coverslip, and imaged at 20× magnification using a Nikon C-DS microscope (Nikon Corporation, Tokyo, Japan).

#### 2.3.5. Creaming Stability Measurement

Emulsion stability during storage was quantified using the creaming index (CI). Ten grams of emulsion were transferred into test tubes (internal diameter 21 mm, height 70 mm) and tightly sealed with plastic caps. After thermal heating, freeze–thaw treatments, or storage, the emulsions were separated into an optically opaque cream layer at the top and a turbid serum layer at the bottom. Emulsion stability was quantified by the creaming index (CI), calculated as

$$\mathrm{CI}=\frac{H_{s}}{H_{e}}\;\times \;100\%$$

With the variables defined below:

*H_s_*: The height of the separated serum phase (aqueous layer);

*H_e_*: The total height of the emulsion column.

#### 2.3.6. Rheological Analysis

Rheology of O/W emulsions was measured on a rotational rheometer (TA Instruments, New Castle, DE, USA) with a cone–plate geometry at 25 °C, with 1 min equilibration before each test.

Steady shear: Apparent viscosity was recorded from 0.1 to 100 s^−1^ to assess shear thinning.

Amplitude sweep: Strain-dependent viscoelasticity at 1 Hz over 0.01–100% strain was used to determine the linear viscoelastic region (LVR).

Frequency sweep: Within the LVR (0.5% strain), dynamic moduli were measured over 1–100 rad/s.

### 2.4. Emulsion Stability

#### 2.4.1. Turbiscan Stability Index (TSI) and Backscattering Variation (ΔBS)

Emulsion stability was assessed using a Turbiscan Lab Expert (Formulaction, Toulouse, France) equipped with a near-infrared light source (λ = 880 nm). Fresh emulsions (20 mL) were transferred into glass sample cells and scanned at 25 °C for 6 h at 10 min intervals. Backscattering (BS) profiles were recorded as a function of sample height and time, with an initial liquid height of 42.5 mm corresponding to 20 mL of emulsion. The Turbiscan Stability Index (TSI) was calculated by the TowerSoft software (version 2.0.0.9, Formulaction S.A., Toulouse, France) for Turbiscan based on the temporal variations in BS intensity, integrating the absolute differences between consecutive scans over the entire effective sample height. TSI values were used as a global indicator to evaluate phase separation and the physical instability of the emulsions.

#### 2.4.2. Thermal Stability Test

Fresh emulsions were heated at 85 °C for 30 min and equilibrated for 24 h at 25 °C. Post-treatment analyses included: (1) optical microscopy (20×); (2) mean droplet size analysis by laser diffraction (Mastersizer 3000, Malvern Instruments Ltd., UK); and (3) measurement of the creaming index.

#### 2.4.3. Freeze–Thaw Stability Test

The freeze–thaw stability was evaluated by freezing at −20 °C followed by 24 h incubation at 25 °C. Post-treatment analyses included: (1) optical microscopy (20×), (2) mean droplet size analysis by laser diffraction (Mastersizer 3000, Malvern Instruments Ltd., UK), and (3) measurement of the creaming index.

#### 2.4.4. Storage Stability Test

Storage stability of the emulsions was evaluated by placing the samples in sealed tubes at 4 °C for 7 days. Post-storage analyses included: (1) optical microscopy (20×), (2) mean droplet size analysis by laser diffraction (Mastersizer 3000, Malvern Instruments Ltd., UK), and (3) measurement of the creaming index.

### 2.5. Statistical Analyses

All experiments were conducted 3 times, and the data were reported as mean ± standard deviation. Statistical analyses were performed using the SPSS 21 geometric software system (SPSS Inc., Chicago, IL, USA). Duncan’s multiple range test was employed to assess significant differences at *p* < 0.05.

## 3. Results and Discussion

### 3.1. Preparation and Characterization of the Ternary Complexes from Grape Pomace

#### 3.1.1. Composition and FT-IR Analysis of the Ternary Complexes

The ternary protein–polysaccharide–phenolic complexes (GPTC) were prepared at both laboratory- and pilot-scale levels. Their compositions are displayed in Table 1. Results showed that the process employed in this study was successfully scaled up from a laboratory scale to 100 kg pilot-scale operations with comparable composition. Compared to laboratory extraction, the composition remained broadly comparable after scaling up to 100 kg. For example, protein content increased, while polysaccharide, ash, and polyphenol content decreased. The differences in sample composition mainly arise from the variations between laboratory-scale equipment and pilot-scale production facilities.

From a practical perspective, the successful production of GPTC at the 100 kg pilot scale demonstrates the technical feasibility of this co-extraction process. The pilot-scale preparation was conducted using standard food-processing unit operations, including raw material pretreatment, protein extraction, centrifugal purification, and freeze-drying. The total technical service cost for the pilot-scale batch was approximately RMB 13,400 (tax included), comprising raw material pretreatment (RMB 1000), protein extraction (RMB 3500), centrifugal purification (RMB 2000), freeze-drying (RMB 4500), and energy and cleaning services (RMB 2400). Considering that grape pomace is a low-cost agro-industrial byproduct and that no external crosslinking agents or complex chemical modifications were required, the overall processing cost remains controllable at the pilot scale, supporting the potential for further industrial implementation.

**Figure 1:**
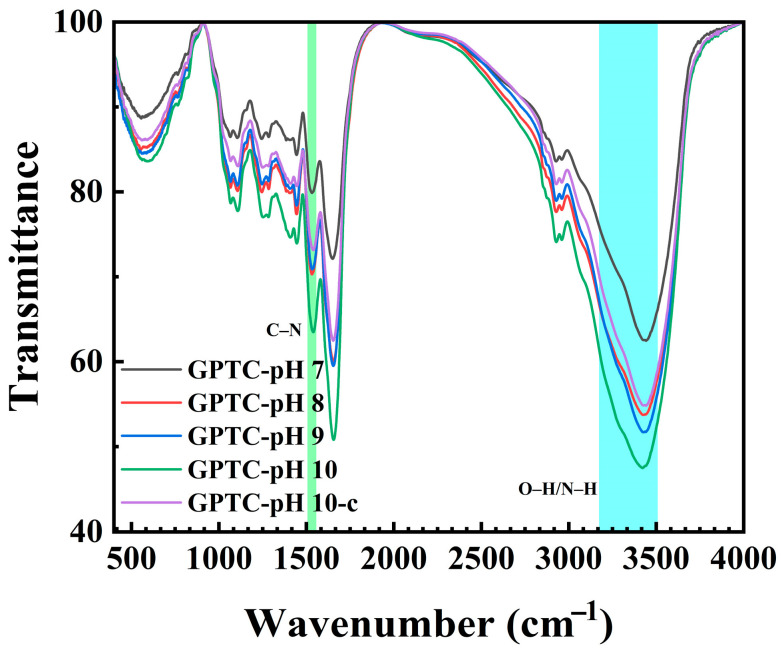
FT-IR spectra of the ternary protein–polysaccharide–polyphenol complexes at different pH values. (In the figure, the green region represents the C–N stretching vibration of amide II, and the blue region corresponds to the broad O–H/N–H stretching vibrations at 3200–3500 cm⁻¹).

To confirm the molecular interactions of proteins, polysaccharides, and polyphenols, the FT-IR spectra of the ternary complexes were conducted, with the results shown in Figure 1. A common characteristic observed in all spectra was a broad O–H/N–H stretching band at 3200–3500 cm^−1^, indicative of hydrogen-bonded hydroxyl groups [18]. The ternary complex at pH 10.0 exhibited an absorption at 3441 cm^−1^, characteristic of O–H/N–H stretching vibrations. As the pH decreased to 9.0, 8.0, and 7.0, this absorption shifted progressively to 3426, 3425, and 3411 cm^−1^, suggesting that hydroxyl groups in polysaccharides and amino groups in proteins gradually formed hydrogen bonds. FT-IR spectra separated the compositions, while the C–N stretching of the amide II absorption was observed at 1544–1530 cm^−1^, indicating that the ternary complexes are rich in protein.

Each item is the dry basis mass fraction determined by an independent method. Due to differences in methodology and uncertainty, the sum may not equal strictly 100%.

#### 3.1.2. Thermal Stability Analysis of the Ternary Complex

**Figure 2:**
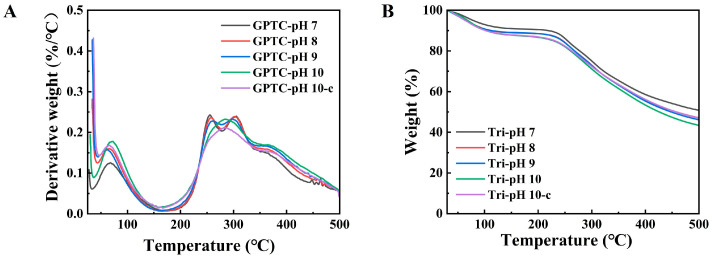
(**A**) Derivative thermogravimetry (DTG, -dW/dT) curves; (**B**) thermogravimetric analysis (TGA) weight loss curves.

Thermal stability is an important quality attribute of food-grade colloidal particles and the emulsions that they stabilize. TGA was employed to investigate the thermal decomposition behavior of GPTC. As shown in Figure 2A,B, it is evident that pH variations do not induce significant alterations in the thermal decomposition behavior of GPTC based on the observation that the thermal decomposition profiles of different samples exhibit convergence.

There are two main mass-loss events; a loss of bound water (70–100 °C) and major component decomposition (240–400 °C). Within the temperature range of 250–350 °C, the main mass loss is attributed to the degradation of polysaccharide and protein components [19]; the main protein chains break at temperatures of 250–300 °C, releasing carbon monoxide (CO) and carbon dioxide (CO_2_). Previous studies on protein–polyphenol complexes have demonstrated that in TGA testing, the complexes exhibit slower decomposition rates and lower ultimate mass loss compared to proteins or polyphenols alone, indicating enhanced thermal stability [20]. Collectively, the protein–polysaccharide–polyphenol ternary complexes presented herein have high the thermal stability. In fact, the stability of multicomponent systems extends beyond thermal stability. Emulsions stabilized by multiple components also demonstrate superior performance in resisting other disturbances. The thermal stability discussed in this section represents only one aspect of this broader stability.

#### 3.1.3. Particle Size and Zeta Potential of the GPTC

The particle size of the complexes critically affects the emulsion droplet size, stability, and other properties. To shed light on the information concerning the size of the particles formed in the above ternary complexes, the average particle sizes and zeta potentials of different complexes were measured at various pH values, and corresponding results were shown in Table 2. The average size of the prepared GPTC increases significantly with increasing pH. The particle size of GPTC at pH 7.0 is 111 nm, whereas its size at pH 10.0 increases to 260 nm, indicating greater association and a higher instability at a higher pH value. This is because under alkaline conditions, which are far from the isoelectric point (pI), proteins and polysaccharide components are denatured. When the pH value decreases, hydrogen bonds reform, resulting in the structure size decreasing accordingly [21]. Mechanistically, as pH increases, ionizable groups on proteins and polysaccharides are deprotonated, which enhances the negative surface charge and weakens hydrogen bonds. This loosens the complexes and promotes association, resulting in an increase in particle size with rising pH. In addition, an increase in pH elevates surface potential and electrostatic repulsion, which in turn modifies particle size [14].

Next, we analyzed the relationship between pH and the particle size or zeta potential of the GPTC samples; the results are displayed in Table 2. It was observed that the particle size of GPTC decreases as pH decreases, accompanied by a decrease in the absolute zeta potential, a similar phenomenon similar to the complexes whey protein isolate (WPI) and peach gum polysaccharides (PGPs) [14]. It is noteworthy that the particle size of these complexes is pH-programmable, meaning it can be selectively tuned by simply choosing the desired pH value. Consequently, by targeting a specific pH level, one can achieve a “stepwise-tuned” output of particle properties. The innovation of this application resides in the use of pH as a programming strategy, with its effects persisting throughout the emulsification process. Particles with a pH of 7.0–8.0 demonstrate faster adsorption and diffusion rates, forming thicker, more cohesive interface layers and producing smaller droplets. In contrast, particles with higher pH values carry greater charges, loosely binding together to form a weaker layer and larger droplets. Adjusting the pH of GPTC from pH 7.0 to pH 10.0 produced a state which was named GPTC-pH 10-c. As shown in Table 2, the particle size and zeta potential of GPTC-pH 10-c differed from those of GPTC-pH 10, suggesting that the interfacial behavior of the samples depends not only on the final environmental pH, but also on the pH conditions experienced during their formation. The pH of the solution environment significantly alters the particle size and surface potential of GPTC particles. Consequently, GPTC-pH 7 could potentially demonstrate greater advantages, which are further examined in subsequent analyses.

#### 3.1.4. Emulsifying Activity (EAI) and Emulsion Stability (ESI) of the GPTC

**Figure 3:**
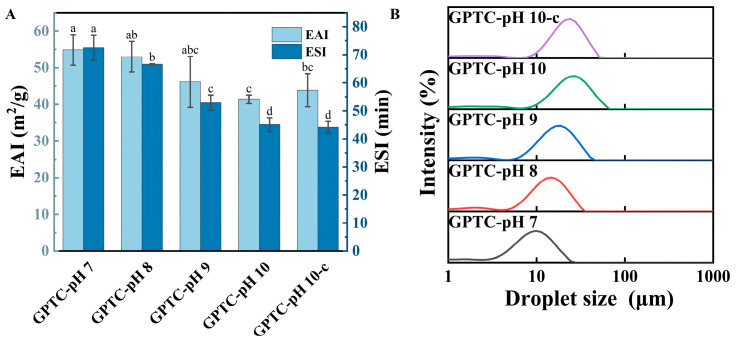
(**A**) Emulsifying activity index (EAI) and emulsion stability index (ESI) of the GPTC. (**B**) Intensity-weighted droplet size distributions of emulsions stabilized by GPTC. Different letters on the bars indicate significant differences among groups (*p* < 0.05).

EAI and ESI are important quantitative indicators for measuring the stability of emulsions. EAI measures the adsorption capacity of emulsifiers at the oil–water interface, while ESI measures the ability of emulsifiers to inhibit phase separation [22]. The EAI and ESI of the GPTC samples at different pH values (Figure 3A) show a decreasing trend with increasing pH. In particular, the emulsifier GPTC at pH 7.0 exhibited the highest emulsification activity index (EAI) of 54.87 m^2^/g, as it rapidly adsorbs at the oil–water interface and effectively reduces interfacial tension. Consistently, the GPTC sample at pH 7.0 also showed the highest emulsion stability index (ESI), indicating its superior ability to stabilize emulsions and effectively suppress phase separation. The EAI value of GPTC gradually decreases over the pH range of 7.0 to 10.0, with GPTC-pH 10c showing a slight increase compared to GPTC-pH 10, indicating the presence of a pH-related memory effect. Meanwhile, the ESI values of GPTC-pH 10c and GPTC-pH 10 are similar, suggesting that the pH memory effect is more pronounced in EAI.

Smaller particles provide a higher specific surface area and faster interfacial spreading, improving EAI and ESI. The observed smaller size under neutral conditions most likely stemmed from enhanced hydrogen bonding, hydrophobic interactions, and π–π interactions among proteins, polysaccharides, and polyphenols; consequently, these stronger interactions facilitate the formation of dense, continuous interfacial films that inhibit droplet coalescence and Ostwald ripening [23], thereby increasing ESI. At higher pH values, particle swelling could reduce interfacial coverage and membrane strength, thereby decreasing EAI and ESI.

The alkaline environment might result in the exposure of the functional groups of the ternary complex, leading to decreased EAI and ESI [24]. However, it was possible that the functional group exposure of GPTC-pH-10-c, which underwent neutralization, was weaker than that of GPTC-pH 10. This phenomenon of “partial adsorption recovery and incomplete viscoelastic recovery” suggests the presence of a “conformation memory effect” within the system, structures that have experienced neutral pH do not fully revert when returned to alkaline conditions. The structural and interfacial behavior of the complexes at an alkaline pH may be influenced by their prior pH conditions, indicating a possible history-dependent response. Similar behavior has been reported for proteins and protein-based complexes, where conformational states formed at specific pH values do not always change in a fully reversible manner upon subsequent pH adjustment [25]. Therefore, complexes stabilized at an intermediate pH could exhibit incomplete structural recovery when returned to alkaline conditions, which may contribute to the observed differences in interfacial performance.

### 3.2. Characterization of GPTC Particle-Stabilized Emulsions

#### 3.2.1. Droplet Size Distribution of Fresh Emulsions and Volume-Weighted Mean Diameter D [4,3]

The size of emulsifier particles largely determines emulsion stability and droplet size [26]. Figure 3B shows the emulsion droplet size distributions produced by different GPTC particles, there is a progressive rightward shift and a broadening as the pH increases. The GPTC emulsion at pH 7.0 is unimodal and tight. In contrast, the GPTC emulsions at pH 8.0 and pH 9.0 shift to larger sizes with slightly broader peaks. The GPTC emulsion at pH 10 shifts further to the right. Compared to the GPTC-pH-10 emulsion sample, the GPTC-pH 10-c emulsion sample displays a reduction in droplet size, agreeing with the above observed results in Table 2. Table 3 lists the volume-weighted average diameter D [4,3] of the prepared emulsions. Larger particles generally lead to larger emulsion droplets under otherwise similar conditions, as the efficiency of interfacial coverage and the way particles pack at the interface vary with particle size. Therefore, the differences in droplet size in Table 3 can be reasonably associated with the differences in particle size shown in Table 2. As shown in Table 3, lowering the pH leads to smaller GPTC emulsion droplet sizes. After pH adjustment, the droplet size of the GPTC-pH 10-c emulsion only partially recovered, as compared to the GPTC-pH 10 emulsion, suggestive of the presence of a pH-driven structural memory effect.

In stable emulsions of ternary complexes, the perspective that proteins contribute surface activity, polysaccharides offer steric hindrance, and polyphenols augment multipoint interactions and stability has been systematically argued and substantiated by experiments [23]. The observed trend—droplet size increases with increasing pH—suggests a direct correlation between the size of the emulsion droplets and that of the complex particles. Under neutral conditions, hydrogen bonding and hydrophobic and π–π interactions among polysaccharides, proteins, and polyphenols drive dense self-assembly and high interfacial coverage. During emulsification, these particles rapidly adsorb to oil droplets, preventing their coalescence into larger droplets. At high pH levels, deprotonation-induced swelling decreases the spreading rate and film strength, resulting in larger droplets. This mechanism aligns with recent reports that ternary complexes regulate interfacial structure to reduce droplet size and enhance stability [27].

#### 3.2.2. Confocal Laser Scanning Microscopy (CLSM) Analyses

**Figure 4:**
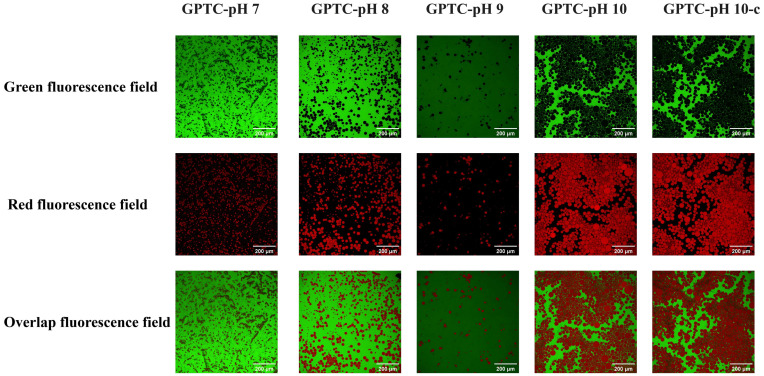
CLSM micrographs of O/W emulsions stabilized by grape pomace ternary complexes. Green channel: FITC-labeled protein/polysaccharide; red channel: Nile red-stained MCT; bottom row: merged images. Scale bar = 100 μm.

To visualize the morphology of these prepared GPTC emulsions, their CLSM images was analyzed in different channels, and the results are shown in Figure 4. Generally, it was observed that emulsion droplet size increases with pH, consistent with the above observed results (Figure 3B). The emulsion sample at pH 7.0 has the smallest and most uniform droplets, while the pH 8.0 emulsion sample has slightly larger droplets.

For the emulsion samples at pH 7.0, continuous, bright, and uniform green fluorescence rings are observed around the droplet interfaces, indicating a relatively thick interfacial coating by the ternary complexes. In the pH 10.0 and pH 10-c emulsion samples, these fluorescence rings are either faint or completely absent, indicating a marked decrease in adsorption and interfacial film thickness under alkaline conditions. This evolution of the interfacial film mirrors the decline of EAI/ESI, suggesting that the adsorption and film formation of GPTC are weakened in alkaline environments. GPTC’s superior performance at pH 7.0 might arise from proteins providing interfacial activity, polysaccharides offering steric hindrance, polyphenols forming multipoint interactions, and hydrophobic/π–π crosslinking, producing dense films with good mechanical integrity. However, at a high pH, polyphenol oxidation and protein structural changes impact the complex architecture and weaken stability [28]. In the CLSM analysis, the rapid adsorption kinetics of GPTC-pH 7.0 at the emulsion droplet interface were visually demonstrated, making it suitable for complex applications in food processing.

#### 3.2.3. Rheology Analyses

**Figure 5:**
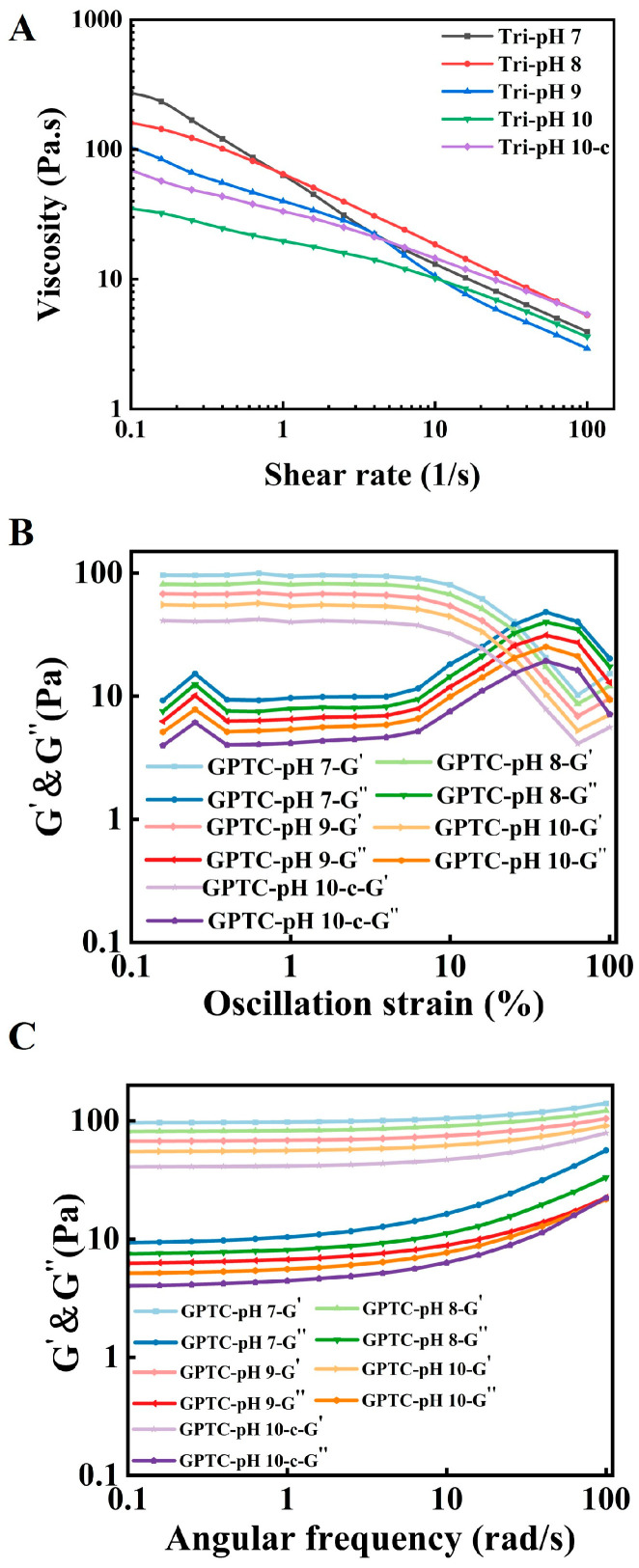
(**A**) Steady shear flow curves (apparent viscosity vs. shear rate, 0.1–100 s^−1^) of the emulsions. (**B**) Amplitude sweep at 1 Hz and 25 °C, showing storage (G′) and loss (G″) moduli versus oscillatory strain for the emulsions. (**C**) Frequency sweep (25 °C, within LVR at γ = 0.5%), showing storage (G′) and loss (G″) moduli.

Rheology provides a direct link between structure and use. As shown in Figure 5A, all emulsions exhibit shear-thinning behavior, with viscosity decreasing at high shear rates [29]. The viscosity–shear rate curves offer a distinct ranking, namely the rate of the GPTC sample treated at pH 7.0 is the fastest, followed by GPTC-pH 8 > GPTC-pH 9 > GPTC-pH 10-c > GPTC-pH 10. This aligns with the general rule that denser interfacial films and smaller droplets at lower pH yield higher zero-shear viscosity and network damping [30]. In amplitude sweeps (Figure 5B), GPTC samples are elastic-dominant (G′ > G″) before yielding, with an LVR of 0.3–2.0%. At medium-to-high strains, G′ and G″ change sharply to reach the yield point, consistent with the yield spectra observed in Pickering or particle-bridged emulsions where particle desorption or network collapse occurs [31].

Within the LVR (Figure 5C), frequency sweeps indicate that G′ exceeds G″ for all samples, demonstrating that all samples display behavior dominated by elasticity. Among these, GPTC shows the highest G′ values at pH 7.0, with minimal frequency dependence, indicating a more robust network between oil droplets, slower recovery, and greater resistance to disturbances [32]. At pH 10.0 or adjusting back to alkaline conditions after pH 7.0, G′ values decrease significantly, reflecting weakened interfacial adsorption and particle bonding.

In summary, the pH value can be used as a critical factor that influences the robustness of the droplet network and the emulsion’s viscoelastic properties. At a suitable pH value, GPTC emulsions exhibit optimal stability and the most pronounced elastic dominance within the linear viscoelastic region (LVR).

#### 3.2.4. Thermal Stability Analyses

**Figure 6:**
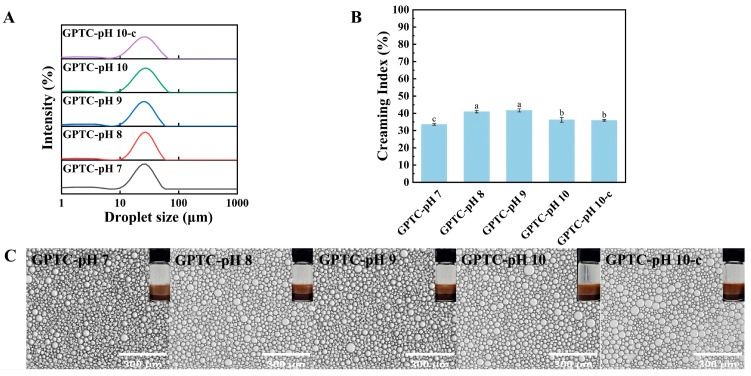
Thermal stability of the emulsions: (**A**) Droplet size distributions upon heating at 85 °C for 30 min, (**B**) creaming index (CI) post heating, and (**C**) optical micrographs of heated emulsions (scale bars = 300 µm). Different letters on the bars indicate significant differences among groups (*p* < 0.05)

Thermal stress simultaneously affects both the interfacial film and droplet network of the emulsion. To this end, we evaluate the thermal stability of the emulsion by examining the droplet size distribution and the stratification observed in the microscopic images. As shown in Figure 6A, after heating, droplet sizes shift to larger values and the distributions broaden. This is expected because higher temperatures can unfold or soften interfacial films, which promote coalescence and aggregation. Prior work reports the same trend; egg-white-protein emulsions heated at 90 °C for 30 min show marked droplet growth, likely due to thermal denaturation of interfacial proteins that facilitates aggregation [33].

Analysis of the creaming index (CI) offers insight into particle aggregation within emulsions and serves as an indicator of their stability. The creaming index of the emulsions after heating was shown in Figure 6B. Among the emulsion samples, GPTC-pH 7 shows the lowest CI value (34%), while GPTC-pH 8, GPTC-pH 9, GPTC-pH 10, and GPTC-pH 10-c fall within the mid-to-high range (45–52%). Thus, the GPTC-pH 7 emulsion sample exhibits only moderate droplet growth and minimal macro-separation, indicating that its interfacial film and droplet network are more robust under heating conditions.

Figure 6C shows a micrograph of the emulsion after heating, where all samples display some degree of flocculation or coagulation. Even so, the GPTC-pH 7 emulsion sample maintains the finest and most uniform droplet clusters, along with the leftmost and narrowest size distribution. The shift toward larger mean diameters with broader distributions is consistent with interfacial film unfolding and increased flocculation [34].

As shown in Table 3, after heating, all emulsion samples show a larger D [4,3]. The mean droplet size exhibited a narrow variation within the range of 24–28 μm. Among all samples, the GPTC-pH 10 sample showed the largest value at 27.57 μm, while the GPTC–pH 9 emulsion sample had the smallest at 24.40 μm. The GPTC–pH 10-c emulsion, at 25.40 μm, lay in the middle, indicating that only a portion of the sample particle size distribution tends toward GPTC-pH 10 emulsion.

#### 3.2.5. Freeze–Thaw Stability Analyses

**Figure 7:**
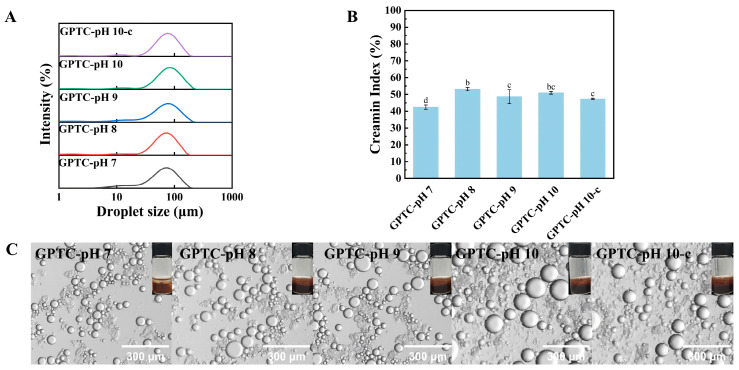
Freeze–thaw stability of the emulsions, (**A**) droplet size distributions after one freeze–thaw cycle (−20 °C for 24 h, then thawed at 25 °C), (**B**) creaming index (CI) after freeze–thaw, and (**C**) optical micrographs of thawed emulsions (scale bars = 300 μm). Different letters on the bars indicate significant differences among groups (*p* < 0.05)

The freeze–thaw stability of these emulsions was also determined, and the results are shown in Figure 7. Generally, freeze–thaw treatment shifts droplet size distributions to larger scales (Figure 7A), leading to elevated CI values (Figure 7B), with large flocs and coalescence spots observed in the micrographs (Figure 7C). Samples including GPTC-pH 8/10/10-c emulsions display an intermediate-to-high CI value (49.96–52.19%) and continuous floc bands; the GPTC-pH 7 emulsion sample has a relatively lower CI and its micrograph still shows finer droplet populations. As compared to the “fresh/heated” results, the GPTC-pH 7 emulsion’s advantage at ambient and elevated temperatures is somewhat diminished under freeze–thaw conditions. This might be because the formation of ice crystals and changes in freezing concentration significantly increase the likelihood of coalescence. During freezing, the crystallization of water causes the continuous phase to contract, squeezing the distribution space of liquid droplets; consequently, droplet spacing decreases and collision frequency increases. Without sufficient viscoelastic interfacial film, polymerization occurs more readily, manifesting after thawing as a rightward shift in the particle size distribution peak and an increase in the CI value.

The GPTC-pH 7 emulsion exhibits optimal performance under fresh conditions, attributed to thicker coatings and a weak gel droplet network. However, freeze–thaw shear forces and concentration variations diminish the spreading ability and inter-droplet linkage of particles/complexes, resulting in its CI no longer demonstrating marked superiority. The GPTC-pH 8/9/10/10-c emulsions, which already possess thinner interfacial films, are more prone to entering a sustained flocculation-coalescence pathway following freeze–thaw, exhibiting higher CI values and coarser microstructures. In short, freeze–thaw destabilization is driven by ice-crystal growth and freeze concentration. These processes damage the interface and disrupt the droplet network. Droplets become compressed and form local bridges. After thawing, this leads to a larger D [4,3] and higher CI.

#### 3.2.6. Storage Stability

**Figure 8:**
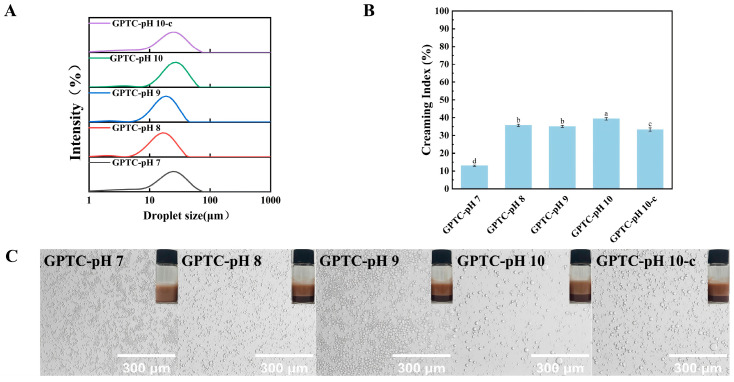
Storage stability of emulsions at 4 °C for 7 days, (**A**) droplet size distributions after storage, (**B**) creaming index (CI) after storage, and (**C**) optical micrographs of stored emulsions (scale bars = 300 μm). Different letters on the bars indicate significant differences among groups (*p* < 0.05).

After storing the emulsions at 4 °C for seven days, the emulsion droplet sizes of various samples were shown in Table 3. The corresponding droplet size distribution curves are presented in Figure 8A, the creaming index (CI) values are shown in Figure 8B, and the macroscopic photographs of emulsions in sample vials together with optical micrographs are displayed in Figure 8C. Following storage, all emulsion samples exhibited varying degrees of phase separation, with increased D [4,3] values. Notably, after seven days of storage at 4 °C, the emulsion stabilized by GPTC-pH 7 particles exhibited the smallest D [4,3] value among all of the stored emulsions, a finding corroborated by its microscopic images. Furthermore, even though all stored emulsions exhibited phase separation, the GPTC-pH 7 emulsion demonstrated the lowest CI value. Under optical microscopy, the GPTC-pH 7 emulsion particles appeared exceptionally fine, further indicating the superiority of this pH environment for stabilizing GPTC particles.

This result indicates that GPTC particles formed under pH 7 conditions achieve optimal performance in interfacial adsorption capacity, wettability, and electrostatic repulsion. This enables effective suppression of phase separation and droplet aggregation during emulsion storage, thereby maintaining high physical stability. In contrast, as pH increases, a change in the surface charge and interfacial behavior of GPTC particles could cause emulsion droplets to aggregate more readily, leading to reduced stability. Although the GPTC-pH 7 emulsion exhibited optimal storage stability, all other samples prepared in this experiment showed varying degrees of phase separation after seven days of storage at 4 °C.

#### 3.2.7. Turbiscan Analyses

TSI (Figure S1) integrates changes in backscattering across the entire height and duration into a global scalar of instability; a smaller TSI indicates greater stability, while a larger TSI indicates lower stability, enabling direct comparison across formulations [33]. From the TSI–time curves and ΔBS–height–time maps of the same Turbiscan run (Figure S2), differences among the GPTC emulsions are clear. The GPTC-pH 7 emulsion shows the lowest TSI in the set. The GPTC-pH 8 emulsion TSI is higher, yet still relatively low. The GPTC-pH 10-c and GPTC-pH 9 emulsions cluster around intermediate TSI values and exhibit similarities to each other. The GPTC-pH 10 emulsion sample exhibits the highest TSI and the lowest stability. The ΔBS maps (Figure S2) reveal dominant mechanisms; both GPTC-pH 10 and GPTC-pH 9 emulsions show early decreases in ΔBS at the bottom with increases at the top, indicating a clearing layer at the bottom and a thickening creamed layer at the top. The GPTC-pH 8 emulsion displays moderate thickening of the top layer and clarification at the bottom, with slower migration compared to pH 9/10. The GPTC-pH 10-c emulsion exhibits a more delayed bottom clarification compared to the GPTC-pH 10 emulsion, suggesting that pH adjustment partially sustains droplet network integrity, yet it remains weaker than that of the low-pH samples. In contrast, the GPTC-pH 7 emulsion sample displays nearly flat ΔBS across most of the column, with only a slight positive peak near the top over time, suggesting nearly constant concentration and droplet size, in line with TSI, which was the lowest. The diagnostic consistency between TSI and ΔBS is high in this dataset [35].

### 3.3. A Comparison of the Present GPTC-Based Pickering Emulsion System and Reported Systems

To provide a clear comparison of previously reported protein–polysaccharide–polyphenol-based Pickering emulsions [11,14,23,27,31], including differences in raw material source, particle type, assembly strategy, chemical modification, scale, and main application, several representative studies are summarized in Table S1. Based on such comparisons, it can be known that all these reported studies first isolated and purified different components from a variety of sources; subsequently, these components were mixed with each other to prepare Pickering emulsions, followed by investigation of their properties. In contrast, this study directly employs natural co-extracts and scales them up in pilot-scale experiments, reflecting a distinct approach to formulation and application orientation.

## 4. Conclusions

This study utilized grape pomace, a byproduct of the wine industry, as raw material to obtain a grape seed protein–polysaccharide–polyphenol complex through co-extraction without additional crosslinking methods. Compared to other pH conditions, GPTC-pH 7 particles formed at pH 7 demonstrated optimal performance. Specifically, GPTC-pH 7 particles exhibited an average size of 111 nm, forming emulsions with a D [4,3] value of 4.8 μm. Rheological testing revealed higher zero-shear viscosity and stronger viscoelastic networks in GPTC-pH 7 emulsions, indicating superior deformation resistance. In simulated storage experiments at 4 °C, the GPTC-pH 7 emulsion exhibited the lowest D [4,3] and CI values during storage. Since the pH 7 environment aligns with most food processing conditions, the material developed in this study could be highly suitable for plant-based protein beverages, protein-fortified foods, and sauce processing in food industry. It also meets food ingredient requirements without necessitating additional crosslinking agents. This work provides a methodological basis for the value-added utilization of agricultural byproducts and the controllable design of food-grade particle-stabilized emulsions, while offering insights for engineering optimization in beverage and nutrient-delivery applications.

## Figures and Tables

**Table 1:** Chemical composition (dry basis) of the ternary complex from grape pomace (lab scale and 100 kg pilot).

Sample	Protein (%)	Polysaccharide (%)	Ash (%)	Polyphenol (%)
**Ternary complex—lab batch**	58.13 ± 0.35 ^b^	27.40 ± 0.04 ^a^	1.36 ± 0.01 ^a^	27.93 ± 0.22 ^a^
**Ternary complex—pilot plant batch**	61.22 ± 0.15 ^a^	22.50 ± 0.11 ^b^	0.88 ± 0.01 ^b^	20.63 ± 0.15 ^b^

Note: Different superscript lowercase letters in the same column indicate statistically significant differences (*p* < 0.05).

**Table 2:** The particle size and zeta potential of the ternary complexes.

Sample	Size (nm)	Zeta Potential (mV)
GPTC-pH 7	111.21 ± 1.73 ^d^	−25.71 ± 1.14 ^a^
GPTC-pH 8	143.22 ± 1.58 ^d^	−29.72 ± 0.83 ^b^
GPTC-pH 9	180.82 ± 2.39 ^c^	−31.34 ± 1.57 ^bc^
GPTC-pH 10	260.24 ± 1.53 ^a^	−35.46 ± 0.69 ^d^
GPTC-pH 10-c	232.60 ± 1.22 ^b^	−32.36 ± 1.28 ^c^

Notes: Different superscript lowercase letters in the same column indicate statistically significant differences (*p* < 0.05).

**Table 3:** Volume-weighted mean droplet diameter D [4,3 (µm) of emulsions: fresh, after thermal heating (85 °C, 30 min), after a freeze–thaw cycle (−20 °C, 24 h), and after 7-day storage at 4 °C.

Sample	Fresh (µm)	Thermal Stability (µm)	Freezing Stability (µm)	Storage Stability (µm)
GPTC-pH 7	9.49 ± 0.00 ^e^	24.87 ± 0.31 ^cd^	78.63 ± 7.81 ^ab^	14.90 ± 0.10 ^d^
GPTC-pH 8	14.33 ± 0.58 ^d^	26.23 ± 0.06 ^b^	76.33 ± 9.76 ^ab^	17.60 ± 0.00 ^b^
GPTC-pH 9	17.77 ± 0.06 ^c^	24.40 ± 0.53 ^d^	66.30 ± 5.41 ^b^	19.40 ± 0.10 ^b^
GPTC-pH 10	25.20 ± 0.00 ^a^	27.57 ± 0.25 ^a^	84.90 ± 4.76 ^a^	29.73 ± 2.75 ^a^
GPTC-pH 10-c	23.03 ± 0.06 ^b^	25.40 ± 0.17 ^c^	77.27 ± 3.00 ^ab^	24.60 ± 0.20 ^c^

Notes: Different superscript lowercase letters in the same column indicate statistical significant differences (*p* < 0.05).

## Data Availability

The original contributions presented in this study are included in the article/Supplementary Materials. Further inquiries can be directed to the corresponding authors.

## Supplementary Materials

The following supporting information can be downloaded at https://www.mdpi.com/article/10.3390/foods15030564/s1, Figure S1. Turbiscan Stability Index (TSI) versus time for emulsions stabilized by ternary complexes prepared at different pH values. Lower TSI indicates greater physical stability. Figure S2. Turbiscan backscattering (BS) profiles versus height and time for emulsions stabilized by the ternary complexes prepared at different pH values. Samples include GPTC-pH 7, GPTC-pH 8, GPTC-pH 9, GPTC-pH 10, and GPTC-pH 10-c. Larger BS changes near the bottom or top of the sample indicate clarification or creaming, respectively. Table S1. Summary of representative studies on protein–polysaccharide–polyphenol-based Pickering emulsions, including raw material source, particle type, assembly strategy, chemical modification, scale, and application focus.
