# Supplementary material for: Exploration of Natural Protein–Polysaccharide–Polyphenol Ternary Complexes from Grape Pomace for Clean-Label Pickering Emulsions Through pH Adjustment
Source: Foods. 2026 Feb 5;15(3):564. doi: 10.3390/foods15030564 (PMC12897015; doi:10.3390/foods15030564)
Supplement: Supplementary file 1 [file foods-15-00564-s001.zip › foods-4102419-supplementary.pdf]

Supplementary Material

for

Exploration of natural protein-polysaccharide-polyphenol ternary complexes from grape pomace for clean-label Pickering emulsions through pH adjustment

Zixuan Du [1], Zhengyang Jia [2], Jianyu Yang [3], Yue Zhao [4], Jiachen Zang [1,], and Guanghua Zhao[1,]*

[1]College of Food Science and Nutritional Engineering, China Agricultural University, Beijing 100083, China;
[2]China Agricultural University-Sichuan Advanced Agricultural & Industrial Institute;
[3]College of Food Science, Northeast Agricultural University, Harbin, Heilongjiang, 150030, China;
[4]Yangtze River Delta Smart Oasis Innovation Center, Zhejiang University, Jiaxing, Zhejiang, 314102, China;
Correspondence:* ***gzhao@cau.edu.cn* *(G.Z.)*

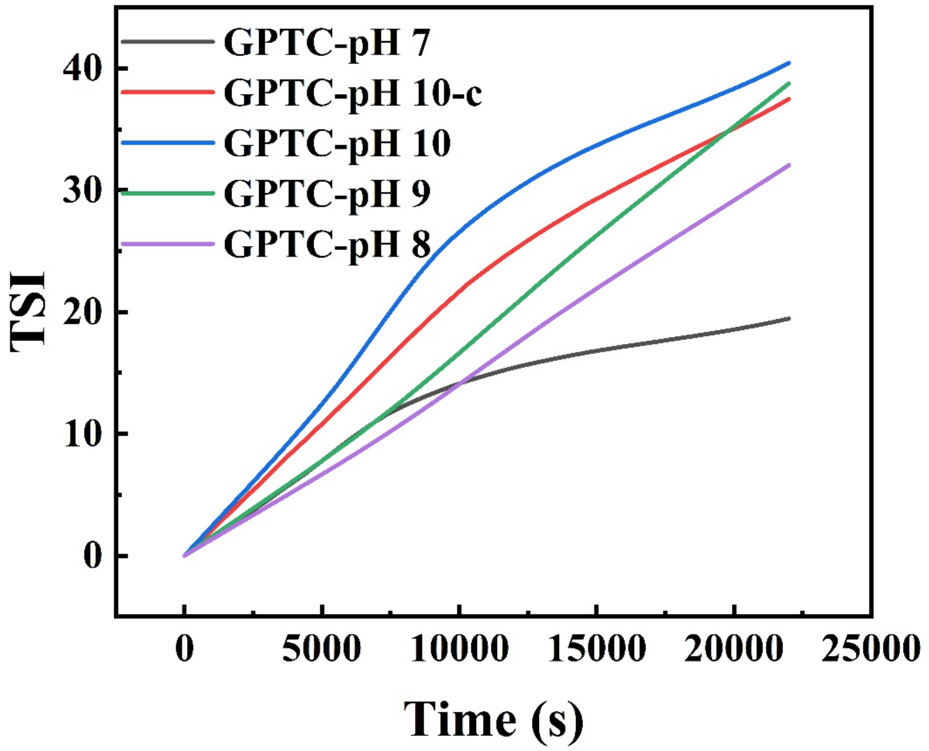

Figure S1. Turbiscan Stability Index (TSI) versus time for emulsions stabilized by ternary complexes prepared at different pH values. Lower TSI indicates greater physical stability.

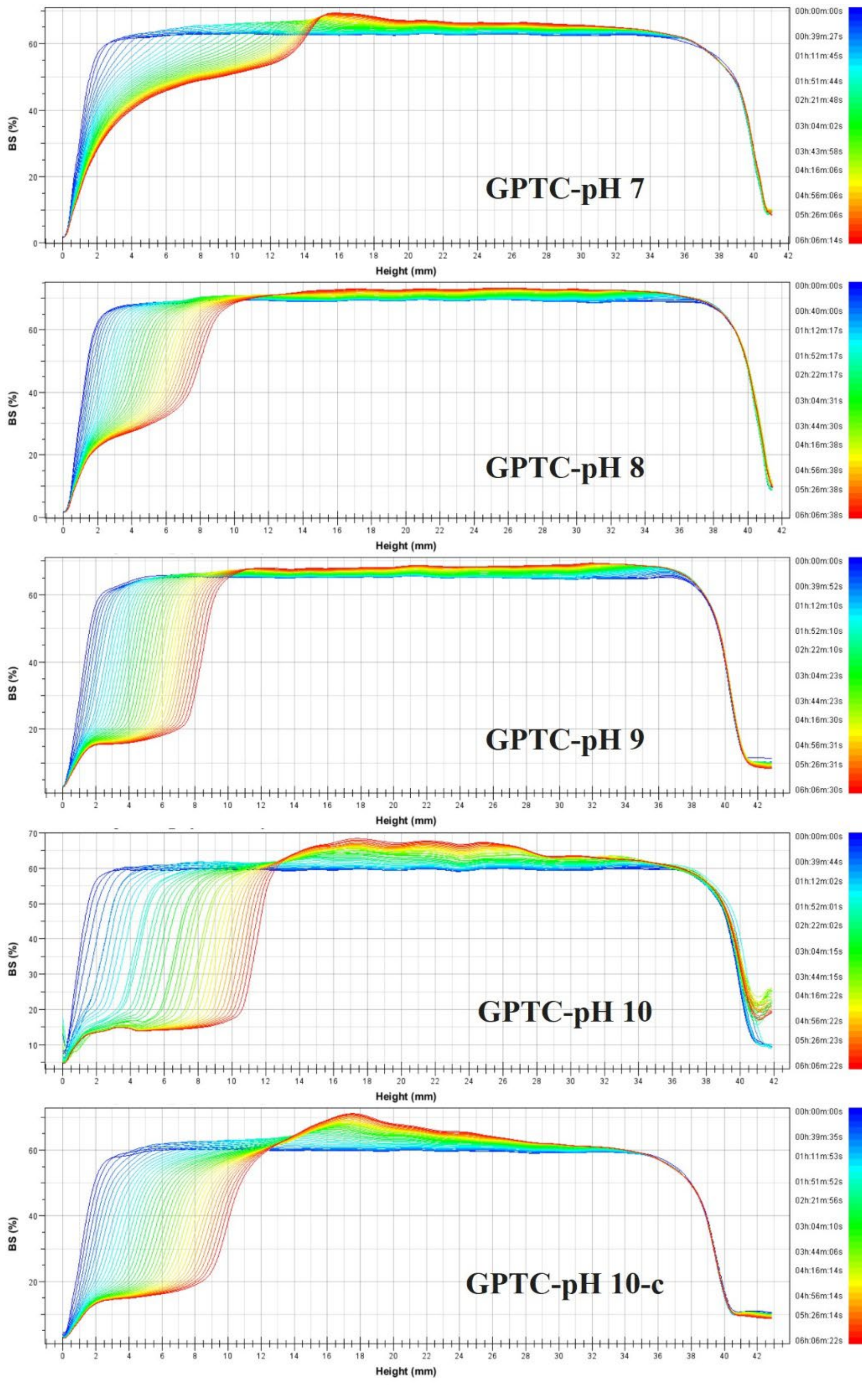

Figure S2. Turbiscan backscattering (BS) profiles versus height and time for emulsions stabilized by the ternary complexes prepared at different pH values. Samples include GPTC-pH 7, GPTC-pH 8, GPTC-pH 9, GPTC-pH 10, and GPTC-pH 10-c. Larger BS changes near the bottom or top of the sample indicate clarification or creaming, respectively.

Table S1. Summary of representative studies on protein–polysaccharide–polyphenol-based Pickering emulsions, including raw material source, particle type, assembly strategy, chemical modification, scale, and application focus.

Study	Raw material source	Particle type	Assembly strategy	External crosslinking or chemical modification	Scale	Main application focus
**Liu et al., 2016** [21]	Purified protein, polysaccharide, polyphenol	Protein–polysaccharide–polyphenol ternary complexes	Recombined ternary complexes from isolated components	Yes (controlled complexation)	Laboratory	Nutraceutical Pickering emulsions and bioaccessibility
**Huang et al., 2024** [25]	Isolated biopolymers	Protein–polyphenol–polysaccharide ternary complexes	Designed stepwise assembly	Yes	Laboratory	Interfacial structure regulation and emulsion stability
**Rayner et al., 2014** [11]	Biomass-derived particles	Biomass-based Pickering particles	Physical particle preparation	No	Laboratory	Food and topical Pickering emulsions

Study	Raw material source	Particle type	Assembly strategy	External crosslinking or chemical modification	Scale	Main application focus
**Huang et al., 2023** [29]	Soy protein and carboxymethyl chitosan	Binary protein–polysaccharide particles	Co-adsorption / electrostatic interaction	No	Laboratory	Rheology and stability of Pickering emulsions
**Zhang et al., 2024 [19]**	Purified protein and polysaccharide	Protein–polysaccharide complexes	Electrostatic complexation	No	Laboratory	Effect of pH on emulsifying performance
